# Access to COVID-19 Vaccines: A New Global Approach

**DOI:** 10.3390/vaccines10111795

**Published:** 2022-10-25

**Authors:** Rabaï Bouderhem

**Affiliations:** College of Law, Prince Mohammad Bin Fahd University, P.O. Box 1664, Al Khobar 31952, Saudi Arabia; rbouderhem@pmu.edu.sa

**Keywords:** COVID-19 pandemic, global public goods, international cooperation, public health, vaccines, WHO

## Abstract

This paper addresses the legal aspects and unprecedented consequences of the Coronavirus disease 2019 (COVID-19) pandemic on the manufacturing and fair access to vaccines. A research literature review allowed us to identify and evaluate the weaknesses of international health law to combat global health crises such as the COVID-19 pandemic. A new paradigm must encourage World Health Organization (WHO) and World Trade Organization (WTO) members to explicitly consider vaccines as global public goods and adopt a new set of legally binding rules for a fair and unrestricted access in times of pandemic. Initiatives and mechanisms such as COVID-19 Vaccine Global Access Facility (COVAX) have been developed to tackle the pandemic and allow developing countries to access vaccines but most were limited and never reached the expected results. The key role played by the WHO in global health policy needs to be strengthened throughout the revision of the International Health Regulations (IHR). Globalization and health are interconnected: WTO members shall revise the Agreement on Trade-Related Aspects of Intellectual Property Rights (TRIPS Agreement) and grant permanent intellectual property (IP) waivers on vaccines in times of pandemic. Our postulation is that vaccines constitute global public goods; their manufacturing and access must be facilitated and guaranteed by specific rules and mechanisms under the supervision of both the WHO and the WTO. It is, therefore, essential to provide the WHO with new powers and attributions to impose coordinated health policies to combat diseases and global crises such as the COVID-19 pandemic.

## 1. Introduction

The COVID-19 pandemic demonstrated that states must opt for greater cooperation throughout compulsory rules and codification especially under the auspices of the WHO [1]. The concept of global public goods [2] with regard to access [3] to vaccines as part of the right to health [4] must be applied to international health law. WHO members need to amend the International Health Regulations (IHR) adopted in 1951 and entered into force for all States Parties in their current form on 11 July 2016 [5]. On 15 June 2022, WTO members only agreed to partially waive intellectual property protections and a narrow exception to an export restriction on COVID-19 vaccines for the duration of five years [6]. Both the IHR and the TRIPS Agreement are interconnected [7]. International health law should be considered as one the cornerstones of interstate relations and help to enhance health systems in all countries [8]. Therefore, sustainable development—one of the objectives of the United Nations—is only feasible and reachable if vaccines are classified as essential medicines and global public goods [9]. COVID-19 vaccines should be considered as essential medicines even though they have been deployed and authorized following emergency procedures [10]. All states shall have access to essential medicines; this right has been claimed by developing countries and officially recognized in 2001 during the WTO’s Doha Conference [11]. Our postulation is that vaccines are essential medicines and global public goods. The theory of global public goods could be applied to COVID-19 vaccines to develop further international cooperation. Indeed, the doctrine traditionally used to consider public health as a global public good, “with a particular emphasis on the control of infectious diseases [12]”. Global public goods—essential medicines such as COVID-19 vaccines and materials—could be implemented in a legally binding treaty signed under the auspices of WHO. UN members do have a fundamental and general obligation to cooperate [13] in situations that may threaten international peace and security including health matters. Here, authors pointed out that cooperation is today very limited. Indeed, it can be described as a “mixed patchwork of achievements and missteps in responding to COVID-19 [that] show powerful nations are not living up to their commitment to solidarity and equity [14]”. The WHO shall be granted adequate powers and prerogatives to initiate coherent answers to global public crises in conformity with the IHR. The goal of this study is to demonstrate that the COVID-19 pandemic created a new paradigm in how states should approach public health crises for greater cooperation. Key players such as the United Nations, WHO and WTO were not able to truly coordinate a global answer in terms of both access to vaccines and health policies despite ambitious mechanisms. As a consequence, reforms of the IHR and the TRIPS Agreements are a necessity to ensure that low- and middle-income countries have access to vaccines and that IP waivers are implemented in times of pandemic.

## 2. Methodology

This paper is based on a research literature review for identifying and evaluating the weaknesses of international health law to combat global health crises such as the COVID-19 pandemic. I searched for articles in English published up to August 2022 on COVID-19 initiatives and responses adopted by the international community, especially the WHO. I reviewed articles in the field of international law, health policy, and public health to analyze how research addresses the question of access to vaccines and the obstacle of IP rights.

Our research focused on publicly available data and laws or policies implemented during the COVID-19 pandemic for providing vaccines to developing countries. I tried to evaluate the efficiency of mechanisms implemented during the COVID-19 pandemic.

The articles were first screened according to title and abstract and then the full texts of eligible articles were evaluated. Moreover, using the same search query, a grey literature research was performed in English on the Google search engine, retrieving articles focusing on access to COVID-19 vaccines with particular attention to policies and laws implemented during the COVID-19 pandemic. I also searched for articles relating to global public goods in order to determine how this concept may be applied to healthcare and vaccines. Finally, I searched WHO’s institutional repositories for additional information. I combined the results from the different sources to outline the framework of COVID-19 responses in the world, emphasizing, where appropriate, the peculiar changes that occurred during the pandemic and their limitations.

Based on data collected and articles reviewed (see Table 1 and Table 2), there is evidence that the low vaccination rate in Africa is directly linked to the lack of international cooperation and the failure of mechanisms implemented by the international community to tackle the COVID-19 pandemic. Indeed, WHO and WTO were not able to coordinate a global response due to dissensions between developed and developing countries relating to IP waivers and access to COVID-19 vaccines. For example, vaccine nationalism did not allow developing countries to have access to COVID-19 vaccines. Additionally, it has been documented that the absence of strong health policies has facilitated the spread of the pandemic in many developing countries.

Research confirms my hypothesis statement following which there is no sufficient interstate cooperation; existing regulations—IHR and TRIPS Agreements—must be amended and consider vaccines as a global public good.

## 3. Limitations of the Existing Mechanisms Implemented to Tackle the COVID-19 Pandemic

**Figure 1:**
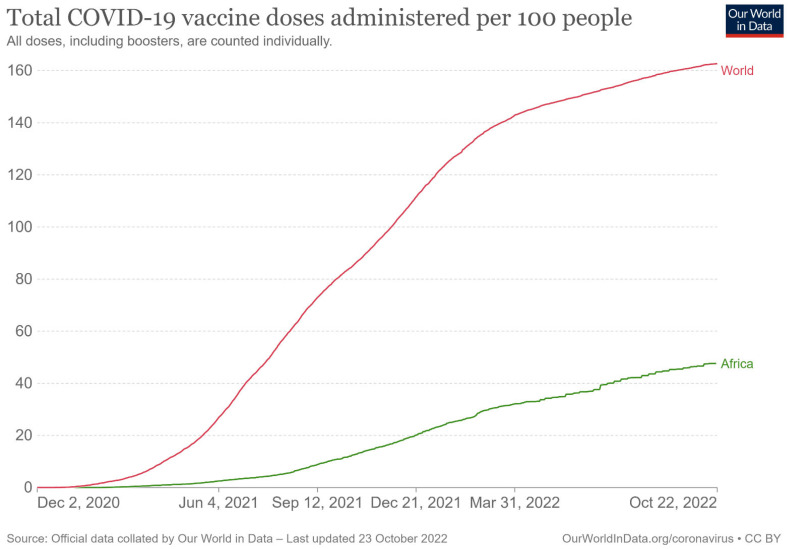
Total COVID-19 vaccine doses administered per 100 people [19].

Research suggests that COVID-19 vaccines will only reach poor countries in 2023 [15]. It has been documented that lower-income countries cannot access vaccines for their populations and pressure is increasing on pharmaceutical companies to allow access to their vaccines [16]. As of 3 May 2022, the vaccination rate in Africa was only 36 doses per 100 individuals compared to 148 doses of the COVID-19 vaccine administered per 100 people in the world [17]. As shown by Elflein [18], the rate of COVID-19 vaccine doses administered worldwide as of 8 September 2022 in low- and middle-income countries (LMICs) was 134.43 per 100 people and only 44.8 per 100 people in Africa compared to 208.39 per 100 people for high income countries. Here, the rate of COVID-19 vaccines administered in Africa has only slightly improved since May 2022 (Figure 1). Different mechanisms were implemented to tackle the COVID-19 pandemic and to facilitate access to vaccines worldwide. However, all these mechanisms were not compulsory: both the WHO and the United Nations relied on interstate cooperation and the goodwill of states.

COVAX [20] and Gavi, the Vaccine Alliance were very ambitious projects [21]. As noted by an author, “the original notion of a global vaccine hub more or less collapsed, and COVAX ended up using a traditional aid-financed approach, which has left lower-income countries wholly at the mercy of wealthy nations and profit-driven companies [22]”. Many developing countries are waiting for their COVAX dose [23] due to several issues (see Table 3). The WHO COVID-19 Technology Access Pool (hereinafter “C-TAP”)—a non-compulsory mechanism—also showed its limitations [24]. C-TAP implementing partners include the Medicines Patent Pool (hereinafter “MPP”) [25], Open COVID Pledge, UN Technology Bank, and Unitaid. The first agreements were signed in October and November 2021. In October 2021, MPP and MSD—the tradename of Merck & Co., Inc. Kenilworth, NJ, USA—signed a voluntary licensing agreement to facilitate affordable access to molnupiravir in 105 low- and middle-income countries [26]. On the 16th of November 2021, Pfizer and MPP announced the signing of a Licensing Agreement for a COVID-19 oral antiviral treatment candidate have expanded access across low- and middle-income countries [27]. Another licensing agreement was signed a few days later on the 23rd of November 2021 by the C-TAP [28]. On the 12th of May 2022, C-TAP and the MPP finalized a licensing agreement with the United States National Institutes of Health (NIH) for the development of several innovative therapeutics, early-stage vaccines and diagnostic tools for COVID-19 [29]. These recent agreements demonstrate that formal and informal mechanisms of interstate cooperation can work efficiently but as I explained a legally binding instrument will provide predictability and legal certainty to low- and middle-income countries (LMICs).

As mentioned in the introduction, the WHO IHR and the TRIPS Agreement are interconnected. Therefore, WTO members need to negotiate new rules under the TRIPS Agreement dealing with the manufacturing of vaccines. All countries should be entitled to access vaccines in times of pandemic. Waivers and suspension of IP rights shall become the general rule. To this effect, the WHO should be working in coordination with the WTO in order to identify situations where the international community is facing a global public health crisis and decide that IP rights will be suspended and waivers applied to developing countries. Indeed, many low- and middle- income countries could not access vaccines due to stringent IP rules. Coordinated responses through mandatory rules will allow the world to recover quickly and turn the page of the COVID-19 pandemic.

Suspending intellectual property rights in times of pandemic will help developing countries to focus not only on their COVID-19 vaccination programs but also on diseases specific to African countries such as tuberculosis or malaria. The United States decided, for example, to support a temporary suspension of vaccine patents [31]. However, many states and the European Union were not willing to waive IP rights—even on a temporary basis—in order to combat the pandemic. They argued that suspending the IP rights of COVID-19 vaccines will dissuade pharmaceutical companies to pursue their efforts in terms of research and development. The European Union (hereinafter “EU”), Germany, Japan and Switzerland were examples of opponents to this idea [32]. Some authors also consider that suspending IP rights should not be encouraged, they believe that pharmaceutical companies are already cooperating and giving access to their vaccines [33]. Here, one can refer to compulsory licensing of pharmaceuticals under the TRIPS Agreement signed in Marrakech in 1994 following the Uruguay Round [34]. WTO Members may indeed allow pharmaceutical firms—located, for instance, in developing countries—to produce a patented product or process without the consent of the patent owner or otherwise using the patent-protected invention itself. Compulsory licensing of pharmaceuticals has been agreed on by WTO Members in 1994. This mechanism is explicitly specified in the TRIPS Agreement. Here, developed countries may adopt such compulsory licensing under the TRIPS Agreement as the generic copy of COVID-19 vaccines will be produced mainly for the domestic markets of developing countries and not for export. The Doha Declaration on TRIPS and Public Health confirms that countries are free to determine the grounds for granting compulsory licenses, and to determine what constitutes a national emergency. The TRIPS Agreement does not specifically list the reasons that might be used to justify compulsory licensing. However, the Doha Declaration on TRIPS and Public Health confirms that countries are free to determine the grounds for granting compulsory licenses, and to determine what constitutes a national emergency. In the present time, the COVID-19 pandemic can be considered as a clear illustration of situations of national emergency. However, as pointed out by some authors the current legal framework of IP rights contained in the WTO Agreements is limited and needs to be improved. Indeed, some authors note that: “the crisis further demonstrates the failure of high-income countries (HICs) to realize the promise they made at the time of the TRIPS negotiations in 1994, that by agreeing to the terms of TRIPS, lower and middle-income countries (LMICs) would benefit from technology transfer and the building of productive capacity [35]”. When analyzing data, the total number of COVID-19 vaccine doses produced by developing countries is not significant. As of 3 March 2021, only South Africa and India were listed among the countries producing COVID-19 vaccines: South Africa only produced 160,000 doses and India 42 million doses [36]. However, Africa is undoubtedly underserved by COVID-19 vaccines. As noted by an author, “17.4[%] of the world population lives [in Africa], yet less than 3% of coronavirus vaccine doses have been given out in Africa [37]”. Other African countries are now producing COVID-19 vaccines. However, as noted by Ekström et al., “there were only 10 vaccine manufacturers across five African countries—Egypt, Morocco, Senegal, South Africa and Tunisia—jointly producing a tiny fraction of the continent’s needs. Most of these countries have undertaken so-called fill and finish packaging and labelling with very limited upstream production of antigen formulations, principally due to a lack of local scientific capacity, along with weaknesses in the commodity supply chain [38]”.

By comparison, with a share of 5.7% of world population, Europe has a share of 8.5% of COVID-19 vaccine doses given in the world as of 25 October 2021. This situation is particularly alarming, and efforts have to be made by the international community: negotiations under the auspices of the WTO should create a legal obligation upon developed countries to be forced to grant licensing of pharmaceuticals to other members in times of pandemic with no limitations in time. The adequate legal tools already exist: the TRIPS Agreement signed in 1994. As mentioned, on 15 June 2022, WTO members only agreed to partially waive intellectual property protections and a narrow exception to an export restriction on COVID-19 vaccines for the duration of five years. However, this solution is only a palliative and does not constitute an adequate global answer to any further global public health crisis. There are no specific rules under this waiver adopted in June 2022 relating to technology transfer or the building of productive capacity in low- and middle- income countries for example. These questions are crucial if the international community wants to facilitate access to COVID-19 vaccines and all essential medicines. WTO members can only agree that the COVID-19 global health crisis impacted massively the world economy [39]. Therefore, the TRIPS Agreement should be amended in order to provide explicit rules in times of pandemic by automatically granting suspensions on IP rights and waivers for essential medicines and vaccines which are global public goods.

## 4. The Theory of Global Public Goods Applied to Vaccines

Healthcare is today recognized as a global public good [40]. In a Report released in 2002, the WHO already recognized and defined the theory of public goods in health as: “any public good whose externalities transcend the boundaries of any individual country [41]”. According to the WHO, the theory of public good applies not only to medicines and/or vaccines but also to the sharing of information, national policies, frameworks, and regulations implemented to tackle a public health crisis. In its Report, WHO also referred to all stakeholders involved in international health and the key role played by: “Some of the most significant agencies involved in international health—and therefore the most likely financers or suppliers of global public goods for health which—are the World Bank, the World Health Organization, bilateral government organizations, nongovernmental organizations (NGOs), and, increasingly, the World Trade Organization (WTO)”. All these key agencies must cooperate and finance efforts towards the elaboration of new vaccines, facilitate research and development or help LMICs in the fair acquisition of vaccines in order to promote vaccine equity [42]. As I noted, health and trade are interconnected: the WHO and the WTO need to coordinate a global response to public health crises such as the COVID-19 pandemic. Economic growth and health should be promoted by states. Information relating to the manufacturing of vaccines must be made available to all states; the same applies to IP rights which could be suspended for vaccines in situations where the WHO will issue a mandatory decision applicable to all state members.

In its 2002 Report, the WHO noted that: “The problem with supplying public goods at the global level is aggravated by the absence of a governing authority that can assign property rights and exact punishment for noncompliant behavior”. In the Basic Documents released in 2020, the WHO acknowledged in Article 21 that: “The Health Assembly shall have authority to adopt regulations concerning: (a) sanitary and quarantine requirements and other procedures designed to prevent the international spread of disease [43]”. Our postulation—shared by the doctrine—is that vaccines developed by pharmaceutical firms necessarily constitute global public goods [44]. This position is today commonly accepted by the scientific community. Hunter et al. note that: “Vaccine inequity is symptomatic of the failure of global governance of the pandemic. The haphazard way in which vaccines are currently distributed must be addressed as part of a global vaccine strategy that includes a system of intellectual-property management, manufacturing, and distribution that ensures that vaccines are made available equitably around the world. Vaccines against pandemic diseases, and the ability to manufacture them, must not be a sequestered asset that maximizes the return to pharmaceutical company executives and shareholders or increases the electability of politicians. They must be a global public good [45]”. From this perspective, it is also conceivable to consider vaccines developed by private pharmaceutical firms and researchers as global public goods as public funds may have served in their elaboration and development. As I pointed out, the challenges faced today by states lie in the absence of legally binding rules to conduct a global answer to public health crises under the WHO and a regulation of IP rights under the WTO to combat vaccine nationalism and stringent IP rules.

European Union authorities also acknowledged that vaccines constitute global public goods. Indeed, in an announcement made on behalf of the European Commission in the early days of the pandemic, Ursula von der Leyen affirmed explicitly that: “The funds raised would not only help kick-start global cooperation, but would also create a truly unique, global public good [46]”. However, the following months proved that states need to improve on several fields such as coordination and cooperation. Most of the mechanisms implemented such as COVAX failed to meet their expectations; vaccine nationalism and lack of transfer of technology for example continue to impact low- and middle-income countries (LMICs). As a consequence, the international community must help to provide new technologies to developing countries and facilitate their implementation, especially in the field of healthcare. Here, again, a parallel could be made with the COVID-19 pandemic and the necessity to regulate more efficiently global health. Some authors studied the theory of global public goods and how it could be applied to COVID-19 vaccines to develop further international cooperation. Indeed, the doctrine traditionally used to consider the concept of global public good as relevant for health, especially for health research and development and communicable disease control [47]. Vaccines and essential medicines as global public goods could be implemented in a legally binding treaty signed under the auspices of the WHO. State members will have obligations to cooperate and assist each other in times of pandemic as the ultimate goal is to prevent any further global health crisis and to allow the world economy to recover.

## 5. The Reform of the IHR and New Coercive Powers for the WHO

Including COVID-19 vaccines in the list of essential medicines will help the international community to combat the spread of the disease. Some authors note that: “voluntary licensing, through mechanisms such as the Medicines Patent Pool (MPP), can allow generic companies in [lower and middle-income countries (LMICs)] to produce and sell essential medicines still under patent for a fraction of the cost of branded versions [48]”. Moreover, “when voluntary licensing cannot be obtained, countries must be encouraged to use existing legal flexibilities in international intellectual property laws to overcome patent barriers that stand in the way of public health”. The WHO regularly advocated for global financial solidarity by establishing the COVID-19 Solidarity Response Fund (SRF) in April 2020 and the external independent WHO Foundation in May 2020. An evaluation of the COVID-19 SRF was commissioned jointly by the United Nations Foundation (UNF) and WHO. The evaluation was conducted between July and November 2021. The evaluation finds that: “the Solidarity Response Fund was highly efficient in terms of its management and function. The Fund set-up and implementation was enacted with speed, purpose and diligence [49]”. A new paradigm is an absolute necessity in order to reshape the WHO IHR (2005) to efficiently combat global public health crises [50]. This new paradigm is the adoption of a global answer throughout the implementation of new binding rules by states to facilitate cooperation and access to essential medicines and vaccines. It is conceivable that the necessity to cooperate under the UN Charter in times of pandemic is a fundamental obligation for all states. The UN International Law Commission listed the obligation to cooperate in situations threatening international peace and security—including health matters—among peremptory norms of jus cogens [51]. Our postulation is that such duty to cooperate necessarily applies in times of global public health crises and must be considered as a mandatory obligation in international health law.

The COVID-19 pandemic highlighted the shortcomings of international health law and especially of the WHO which has no enforcement powers (see Table 4). The limitations of the WHO have been addressed by researchers [52]. Moreover, the WHO has been criticized by commentators for its “rather restrained role in creating new norms under its Constitution [53]”. Despite the fact that the WHO has a recognized expertise and a constitutional mandate to tackle pandemics and global public health crises, it only provides soft rules such as guidelines and recommendations to states parties. As noted by Gostin et al., “the WHO’s most salient normative activity has been to create ‘soft’ standards underpinned by science, ethics, and human rights. Although not binding, soft norms are influential, particularly at the national level where they can be incorporated into legislation, regulation, or guidelines [54]”. In international law, soft rules are necessary as they allow the international community to reach a consensus on certain matters, and they simplify the adoption of a formal treaty, for example. However, strong answers are necessary in times of pandemic as many states parties may decide not to actively cooperate with other members or international organizations. Indeed, vaccine nationalism emerged and low-income countries are still facing challenges to access vaccines and other essential medicines. Consequently, the WHO should be granted a real normative power throughout a set of legally binding rules. The IHR [55] have been considered as a fundamental development in international law [56]. These regulations are an existing legal tool that can provide the WHO outstanding attributions in times of pandemic. Article 2 IHR, which is related to purposes and scope of the WHO—one of the most important provisions—states that “the purpose and scope of these Regulations are to prevent, protect against, control and provide a public health response to the international spread of disease in ways that are commensurate with and restricted to public health risks, and which avoid unnecessary interference with international traffic and trade”. It is also worth referring to Part II of the IHR related to Information and Public Health Response, especially Articles 12 and 13. These provisions give important powers to the WHO but there are no enforcement powers in situations where state members refuse to cooperate nor coercive measures that can be taken against such states.

During the UN Conference on the Human Environment, states parties acknowledged that international economic law and development are intrinsically linked [59]. The duty to cooperate in health matters can be considered as a principle of international law and a fundamental component of the new international economic order. Our postulation is that such solution should be applied to international health law. UN sustainable development goals (UN SDGs) explicitly refer to good health and well-being [60]. It is undeniable that good health and better health systems cannot be achieved without a fair and equal access to essential medicines and vaccines in times of pandemic.

## 6. Conclusions

Undoubtedly, the WHO played a crucial role in the COVID-19 pandemic even though its powers are limited. The consequences of this global public health crisis must incite states to increase cooperation and grant the WHO new coercive powers in order to be able to coordinate a global answer and facilitate access to vaccines which are necessarily essential medicines and global public goods. As explained, the COVID-19 pandemic had unprecedented effects on both global health and trade. It has been acknowledged that economic growth and healthcare are among the UN SDGs. Therefore, the international community needs to adopt new legally binding rules in order to be able to coordinate a global answer in situations of global health crises. The WHO is a universal international organization which has the capabilities to provide adequate public health policies. The new international economic order demonstrated that states cannot limit themselves to national and domestic policies to combat threats to public health such as the COVID-19 pandemic. International cooperation—a duty for states in international law—should be at the center of international health law. All states should have access to vaccines developed by pharmaceutical firms. Legal instruments do exist, and reforms are possible if states realize their interdependence and allow vaccines to be manufactured and distributed with no restrictions. Recently, the WHO stated that isolated national health policies such as booster programs will only exacerbate vaccine inequity [61]. Efforts have been made by WTO members in June 2022 when the General Assembly of the WTO accepted to waive IP rights on COVID-19 vaccines although such waiver is only temporary and limited to five years. This step towards a global answer allows us to believe that successful negotiations under the auspices of the WHO are possible and that state members could agree to revise the IHR and classify vaccines as global public goods for which access would be facilitated in times of pandemic. On the other hand, WTO members should also revise the TRIPS Agreement and modify IP rules in times of pandemic.

## Figures and Tables

**Table 1:** Quantitative data used in the study.

Quantitative Data by Type
Number of peer-reviewed articles consulted: 130
Number of peer-reviewed articles cited in the study: 30
Data/Charts cited from STATISTA: 7
Reports cited from WHO: 14
Reports cited from WTO: 6
Reports cited from the UN: 4
Report cited from the Organization for Economic Co-operation and Development (OECD): 1
Declaration cited from the European Union Commission: 1
Private institute cited (Center for Global Development; IOD PARC): 2
Articles cited from newspapers/press releases: 3

**Table 2:** Overview of the reviewed sources.

Authors	Country	Purpose	Type of Source	Summary
Maxwell J. Smith et al.	USA	Demonstrate that vaccines deployed and authorized following emergency procedures are also essential medicines	Research/Article	COVID-19 vaccines should be considered essential medicines.
Alexandra Phelan et al.	USA, UK, Australia, Kenya, China	Demonstrate that current regulations can enable but also be barriers to an equitable and global access to vaccines	Research/Article	International agreements under the WHO are necessary to ensure a fair and equitable access to vaccines, previous public health crises have shown that developing countries cannot secure sufficient doses for their populations.
Michaela S. Halpern	UK	Demonstrate that states must cooperate actively to tackle the COVID-19 pandemic	Research/Article	States do have an obligation to cooperate not only in matters that may threaten international peace and security, but also in health matters.
Jesse B. Bump et al.	USA, Norway, Sweden, UK	Demonstrate that states must cooperate actively to tackle the COVID-19 pandemic	Research/Article	WHO’s mandate to tackle global public health crises is limited. Greater interstate cooperation is necessary to combat vaccine nationalism. The WHO’s authority must be strengthened.
T. V. Padma	India	Demonstrate the limitations of all mechanisms implemented to help developing countries in accessing COVID-19 vaccines	Research/Article	Failure of the international community as many developing countries do not have access to COVID-19 vaccines yet.
Amy Maxmen	USA	Developing countries do not have access to COVID-19 vaccines due to IP rights and technological issues	Research/Article	The author advocates for a fair distribution of vaccines and points out the limitations of existing regulations: indeed, pharmaceutical firms opposed IP waivers.
Anne Mills	UK	The author applies the theory of global public goods for better access to healthcare	Research/Article	By analogy, vaccines developed in times of pandemic have to be classified as global public goods and made available to all.

**Table 3:** Problems faced by LMICs.

Typical Problems Faced by LMICs to Access COVID-19 Vaccines
1. Technology transfer
2. The building of productive capacity
3. Vaccine nationalism
4. Financial cost: LMICs have to sign agreements with international organizations such as the International Monetary Fund (IMF) and the World Bank (WB) to finance acquisition of vaccines and essential medicines
5. Stringent IP rules and absence of real IP waivers for LMICs
6. Lack of cold chains and vaccination sites that can maintain the Pfizer and Moderna vaccines at ultra-cold temperature for safety and efficiency
7. Lack of infrastructure such as transportation systems
8. The development of vaccines based on mRNA requires colder environments for stability
9. Ability to implement strong vaccination programs [30]

**Table 4:** WHO’s normative powers.

Existing Powers and Attributions of the WHO	Expected Powers and Attributions Based on a Reform of the IHR
Providing leadership on matters critical to health and engaging in partnerships where joint action is needed	Cooperating with key agencies—IMF, WB, WTO—but also the private sector through public-private partnerships (PPPs), creation of Task Forces for example
Shaping the research agenda and stimulating the generation, translation and dissemination of valuable knowledge	Directing states parties on R&D and facilitating the sharing of information through compulsory mechanisms
Setting norms and standards and promoting and monitoring their implementation	Adopting legally binding rules such as health policies to control the spread of viruses in times of pandemic
Articulating ethical and evidence-based policy options	Reforming and updating existing global regulations for infectious disease control, namely the IHR [57]
Providing technical support, catalyzing change, and building sustainable institutional capacity	Granting the WHO inspection, policing or enforcement powers against its member States
Monitoring the health situation and assessing health trends	Adopting coercive measures if states parties do not respond to a public health emergency of international concern (PHEIC)—formal declarations issued by the WHO [58]

## Data Availability

All data generated or analyzed during this study are publicly available.
